# “Patient-Specific” Cost and Quality Value Comparison of Endoscopic Carpal Tunnel Release in Two Surgical Settings

**DOI:** 10.1016/j.jhsg.2025.100802

**Published:** 2025-08-13

**Authors:** Alex Lindahl, Trae Hill, Emily Mazier, Meet Patel, Geoffrey Kahn, Yueren Zhou, Charles S. Day

**Affiliations:** ∗ Oakland University William Beaumont School of Medicine; † Henry Ford Health System; ‡ Wayne State University School of Medicine; § Michigan State University College of Human Medicine

**Keywords:** Endoscopic carpal tunnel release, Cost-effectiveness, CTR, Time-driven activity-based costing, Value

## Abstract

**Purpose:**

Prior studies have demonstrated that transitioning surgeries from a hospital outpatient department (HOPD) to an ambulatory surgical center (ASC) lowers costs. With 500,000 carpal tunnel release (CTR) surgeries annually, CTR offers an opportunity to determine the value of one of the most performed upper-extremity surgeries. We aim to quantify the value of an endoscopic CTR in a HOPD compared to an ASC by analyzing differences in costs and patient outcomes. We hypothesize the ASC will provide greater value by lowering costs while maintaining patient outcomes.

**Methods:**

Total costs were comprised of time-driven activity-based labor costs (TDABC), activity-based supply costs, and claims-based facility costs. Differences in preoperative Patient-Reported Outcome Measure (PROM) Information System Upper Extremity (UE) and Pain Interference (PI) scores and 3-month postoperative PROM Information System UE and PI scores were calculated to determine PROM Information System Quality-Adjusted Life Years (QALY_UE/PI_). Total costs were divided by QALYs for each PROM to calculate the Value_UE/PI_ of each cohort. The magnitude of the difference in value between cohorts was elucidated by calculating incremental cost-effectiveness ratios.

**Results:**

A total of 25 patients comprised each cohort. The ASC generated 28% lower costs compared to the HOPD ($3,370.73 ± $128.80 vs $4,654.75 ± $140.19). Average QALY_UE_ and QALY_PI_ gain was not significantly greater for patients at the ASC compared to the HOPD (QALY_UE_ 1.06 vs 0.89; QALY_PI_ 1.22 vs 0.92). The ASC demonstrated 40% to 45% greater value, represented by a lower cost/QALYs, compared to the HOPD (Value_UE_ $3,168.81/QALY vs $5,242.78/QALY, Value_PI_ $2,759.90/QALY vs $5,038.04/QALY).

**Conclusions:**

We observed between 40% to 45% greater value by performing CTRs in the ASC. Although ASCs lowered costs by 28%, costs alone do not fully explain the value differential. Patient-reported outcomes serve a valuable role in providing a holistic picture of the value being delivered to patients. Providers can use this information to guide patient decision-making regarding operative treatment options for carpal tunnel syndrome.

**Type of study/level of evidence:**

Economic/Decision Analysis II.

Health care costs, as a percentage of gross domestic product, in the United States have consistently ranked the highest among developed nations since the early 1980s.^1^ Moreover, there is controversy over whether the quality of care being delivered is justified by these expenditures.^1^ The Centers for Medicare & Medicaid Services have spearheaded a multitude of value-based reimbursement initiatives,^2^^,^^3^ emphasizing the critical significance of value-based health care, defined in terms of the cost required to elicit a change in patient outcomes.^4^^,^^5^ One initiative, the creation of ambulatory surgical centers (ASCs), offers an alternative to traditional hospital-based care (HOPD).^6^ The Department of Health and Human Services identified a cost savings of nearly $7 billion between 2007 and 2011 associated with using ASCs over HOPDs.^7^ Furthermore, established benefits of conducting surgeries in an ASC environment include lower rates of readmissions and postsurgical complications compared to HOPDs.^8^^,^^9^ Based on this, one would assume ASCs would provide greater value when evaluating costs and outcomes simultaneously; however, this has not been demonstrated in the literature.

Although a plethora of studies have been conducted within orthopedics to assess cost-efficient alternatives of health care delivery,^10^, ^11^, ^12^, ^13^ few have also included assessments of the quality of care using patient-reported outcomes.^14^^,^^15^ In these few reports, even fewer examined value as defined above.^16^ Moreover, a gross lack of standardization for evaluating the quality of care in conjunction with the limitations of many common cost accounting methodologies has obscured the ability to differentiate real-world patient-centered value among available treatment options,^16^ defined as the difference between the proportion of costs to the change in outcomes of the two different interventions.^16^

One opportunity to grasp a better understanding of health care value would be to evaluate carpal tunnel syndrome, which affects between 1% to 5% of the general United States population, with a peak incidence near 50 years of age.^17^ Cost analyses comparing carpal tunnel release (CTR) surgeries performed in an HOPD to an ASC using robust accounting methodologies have demonstrated considerable cost savings when opting for an ASC.^18^ Considering this, to our knowledge, no study has incorporated quality of care using patient-reported outcomes, in conjunction with accurate costing methodologies, to assess differences in the value of performing a CTR in an ASC and HOPD.

Therefore, the purpose of our study is to compare the patient-level value of performing a CTR in HOPD and ASC settings by totaling the comprehensive cost and using the Patient-Reported Outcome Measure Information System (PROMIS).^19^ We hypothesize that surgeries performed in the ASC will generate significantly more value compared to analogous surgeries performed in a hospital setting. Our secondary hypothesis is that patient-reported outcomes will be comparable between the surgical venues.

## Materials and Methods

Patients were recruited from a single physician’s practice in a multihospital, tertiary care, academic institution in the Midwest. Prospective data were collected from patients undergoing an endoscopic CTR in either the HOPD or ASC setting. Patients <18 years old, those undergoing combined procedures, and those undergoing CTR in a different surgical setting were excluded.

A detailed step-by-step process map was created for each cohort and converged because of observed overlap of workflows following direct observation of personnel involved in providing care on the date of service, from the time of arrival until discharge.^20^ A digital stopwatch was used to capture the time allocated by each staff member.

The total cost of each visit was broken down into four categories. Direct variable labor (DVL) costs were derived from the salary of personnel providing care on the date of service, based on the length of time each member spent with the patient.^21^ Direct variable supply (DVS) comprised the equipment used during the visit. Direct fixed (DF) costs were nonvariable costs associated with providing services on the date of surgery, such as building equipment, utilities, and facility rent.^21^ Indirect costs correspond to costs not directly related to patient care but nonetheless are essential to allow the patient-physician interaction to occur.^22^ Examples include marketing and administrative costs.^22^ All costs associated with providing services on the day of the visit were factored into total direct costs (DF, DVL, and DVS).

Using the TDABC methodology, DVL costs were calculated by multiplying the average time each personnel spent completing steps of the process map by their per-minute cost.^21^ Average annual personnel salaries from the study’s institutions were divided by their working capacity to calculate a cost-per-minute unit.^23^ We approximated an 80% theoretical working capacity to account for personnel inefficiencies, such as breaks and other activities not related to patient care.^24^^,^^25^ Additionally, 4 weeks per year were subtracted from an individual’s working capacity for assumed vacation.^24^^,^^25^ The DVL cost for each surgery was calculated as the sum of all personnel’s labor costs.^20^

The DVS costs were aggregated using activity-based costing (ABC) for each piece of equipment used during the patient’s visit. Additionally, because of the uncertainty of TDABC to calculate DF costs precisely, we obtained these values using validated hospital accounting claims-based technical fee data (EPSi; Allscripts, Libertyville, IL), by examining the allocation of rent for the payer’s technical claims cost of that case, which was calculated via hospital accounting department methodology ((EPSi; Allscripts, Libertyville, IL).^21^ Finally, indirect costs were estimated as a fixed proportion (40%) of the total direct costs. This rate was determined using the average ratio of indirect to total direct costs for an HOPD and ASC procedure, a methodology described in previous TDABC studies.^21^^,^^24^^,^^26^

To quantify the quality of care, PROMIS Upper Extremity (UE) Computer Adaptive Test v2.0 is a general patient-reported outcome measure to assess function of the upper extremity, with higher scores representing greater function (0–100).^27^ The PROMIS Pain Interference (PI) Computer Adaptive Test v1.1 differs in that it assesses the degree to which pain hinders a patient’s ability to engage in physical activities, with lower scores representing decreased hindrance (0–-100).^27^ Both measures have demonstrated acceptable responsiveness for evaluating changes in a patient’s clinical condition following CTR.^27^ The PROMIS UE and PI data were collected before surgery and at the 3-month postoperative visit.

For each metric, differences in preoperative PROMIS scores and 3-month follow-up PROMIS scores were calculated for each patient and averaged to compare between surgical settings.^28^ For ease of interpretation, these numbers were transformed to represent improvement in their respective metric; a positive change in PROMIS UE and a negative change in PROMIS PI were denoted to have a positive utility. These values were then multiplied by the estimated number of life-years remaining for each patient based on their sex, ethnicity, and age at the time of surgery,^29^ to determine PROMIS Quality-Adjusted Life Years (QALY) for each outcome metric. The total cost was then divided by the total QALYs to determine the average Value_UE_ and Value_PI_ of each surgical modality,^30^ whereby lower Value_UE/PI_ equates to greater cost-effectiveness. These numbers were then compared between surgical modalities to determine the incremental cost-effectiveness ratio (ICER) for each outcome metric.^30^ Separate incremental cost-effectiveness planes were generated for PROMIS UE and PROMIS PI results.

Cost and outcome data were bootstrapped 1,000 times to construct empirical 95% confidence intervals for the Value_UE/PI_ and ICER calculations.^31^ Cost-effectiveness acceptability curves (CEAC) were constructed for each outcome metric. CEACs represent the probability of each cohort being cost-effective, relative to the other cohort, for a broad range of willingness-to-pay threshold values. These probabilities were determined by the relative proportion of bootstrapped patients per cohort whose Value_UE/PI_ was below a given threshold, where greater relative proportions translate to increased probability of being cost-effective compared to the other cohort.

Power analysis demonstrated a minimum sample size of 25 patients per cohort to elucidate a hypothesized 20% difference in value per group. Student’s *t*-test with significance defined as an α < .05 was used to evaluate for significant differences in cost and outcomes between cohorts.

Institutional review board approval was sought and granted because of the nature of the data collected (Henry Ford Health IRB #13841). No funding was required for this study.

## Results

**Figure 1:**
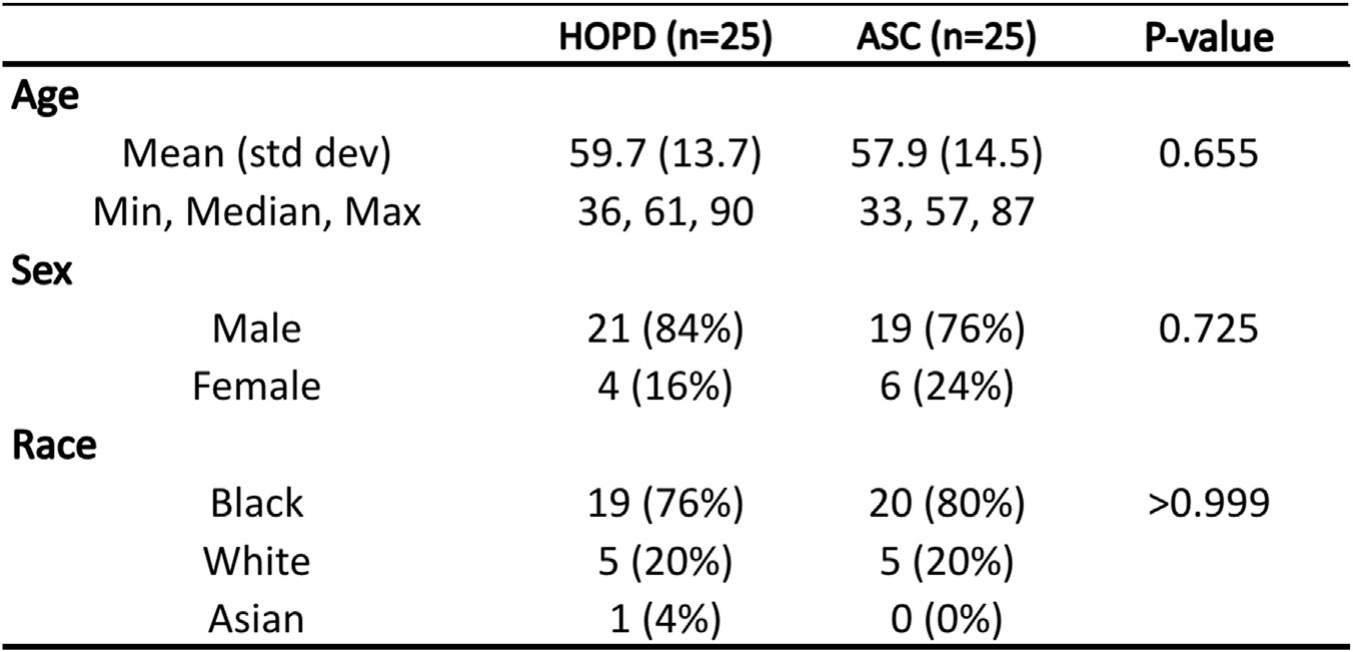
Patient demographics of each cohort are categorized by age (years), sex, and race. n = 25 per surgical modality. Significance was defined as *P* < .05 using the Wilcoxon and Fisher exact test.

**Figure 2:**
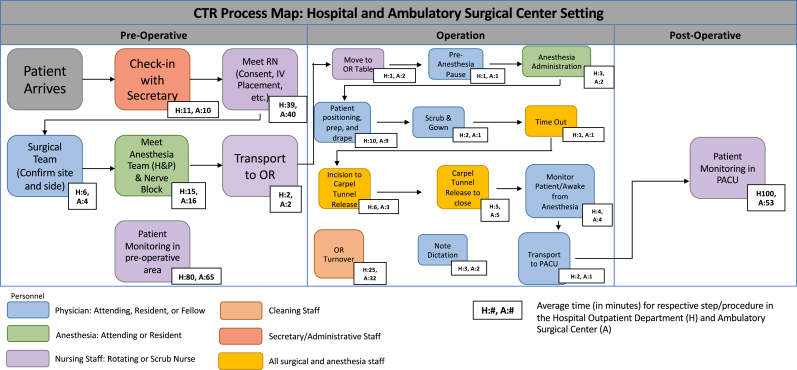
Process maps of HOPD and ASC CTR surgery. The average time to complete each step of the procedure is recorded on the map. n = 25 per surgical modality.

**Figure 3:**
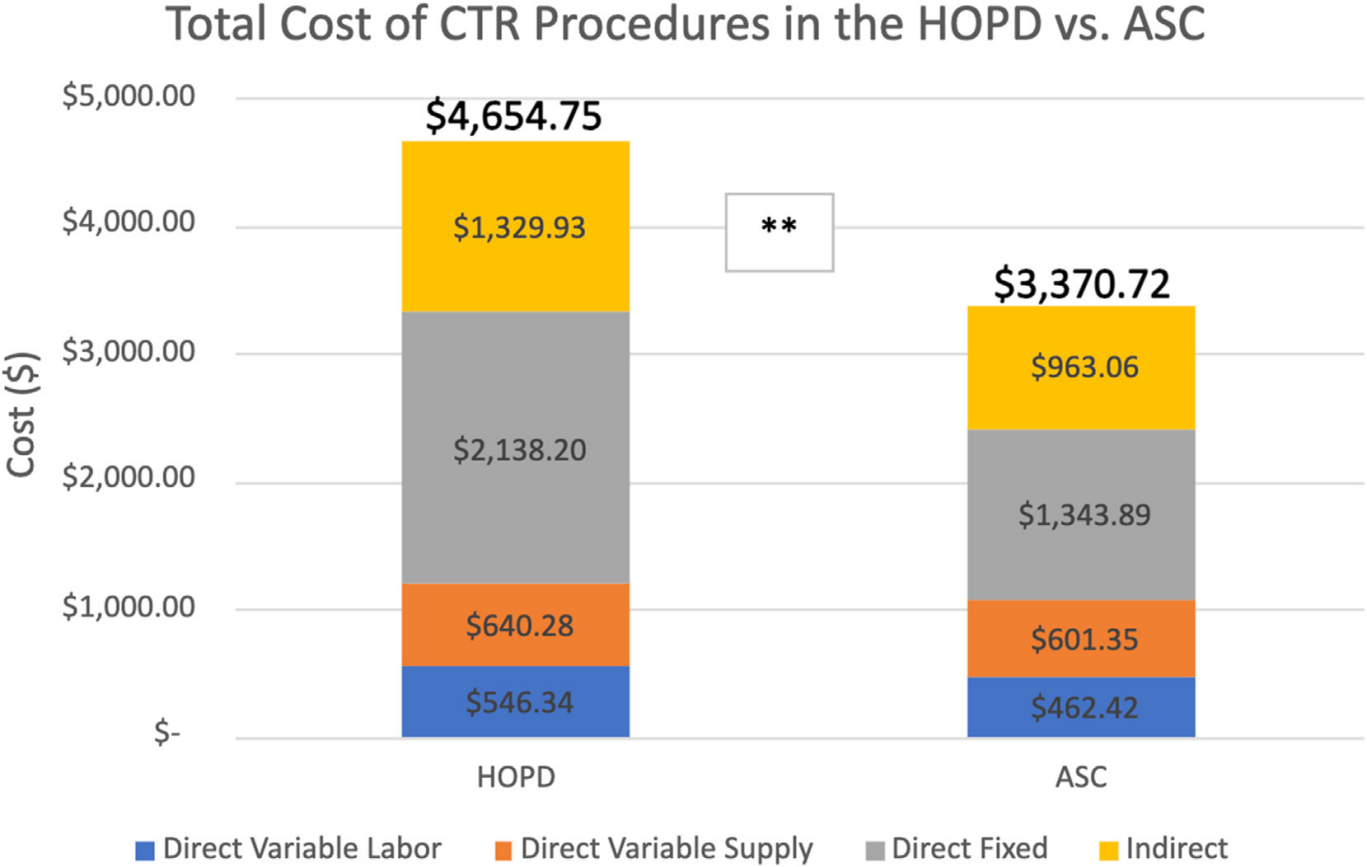
Comparison of the HOPD and ASC CTR total costs according to the TDABC methodology. The total cost is broken down into DVL, DVS, DF, and indirect costs. n = 25 per surgical modality. ∗*P* < .05 and ∗∗*P* < .01 as determined by Mann-Whitney *U* test.

Patient demographics are presented in Figure 1. No significant differences were observed between cohorts based on age, sex, or race. The process map combining workflows of the HOPD and ASC is presented in Figure 2. The average total cost of performing an endoscopic CTR in the HOPD and ASC was $4,654.75 ± $140.19 and $3,370.73 ± $128.80, respectively (*P <* .001), as illustrated in Figure 3. The HOPD costs were composed of DVL costs of $546.34 ± $82.90, DVS costs of $640.28 ± $73.08, DF costs of $2,138.20, and indirect costs of $1,329.93. ASC costs were categorized into DVL costs of $462.42 ± $70.70, DVS costs of $601.35 ± $44.53, DF costs of $1,343.89, and indirect costs of $963.06.

Overall, DVL costs decreased in the ASC by $83.92 ($546.34 ± $82.90 HOPD, $462.42 ± $70.70 ASC, *P <* .001*).* Two key personnel had a significantly lower average cost in the ASC: the senior hand surgeon ($138.60 ± $39.20 HOPD, $104.31 ± $31.07 ASC, *P =* .001 and the registered nurse ($150.44 ± $26.51 HOPD, $108.00 ± $18.84 ASC, *P* < .001*).*

**Figure 4:**
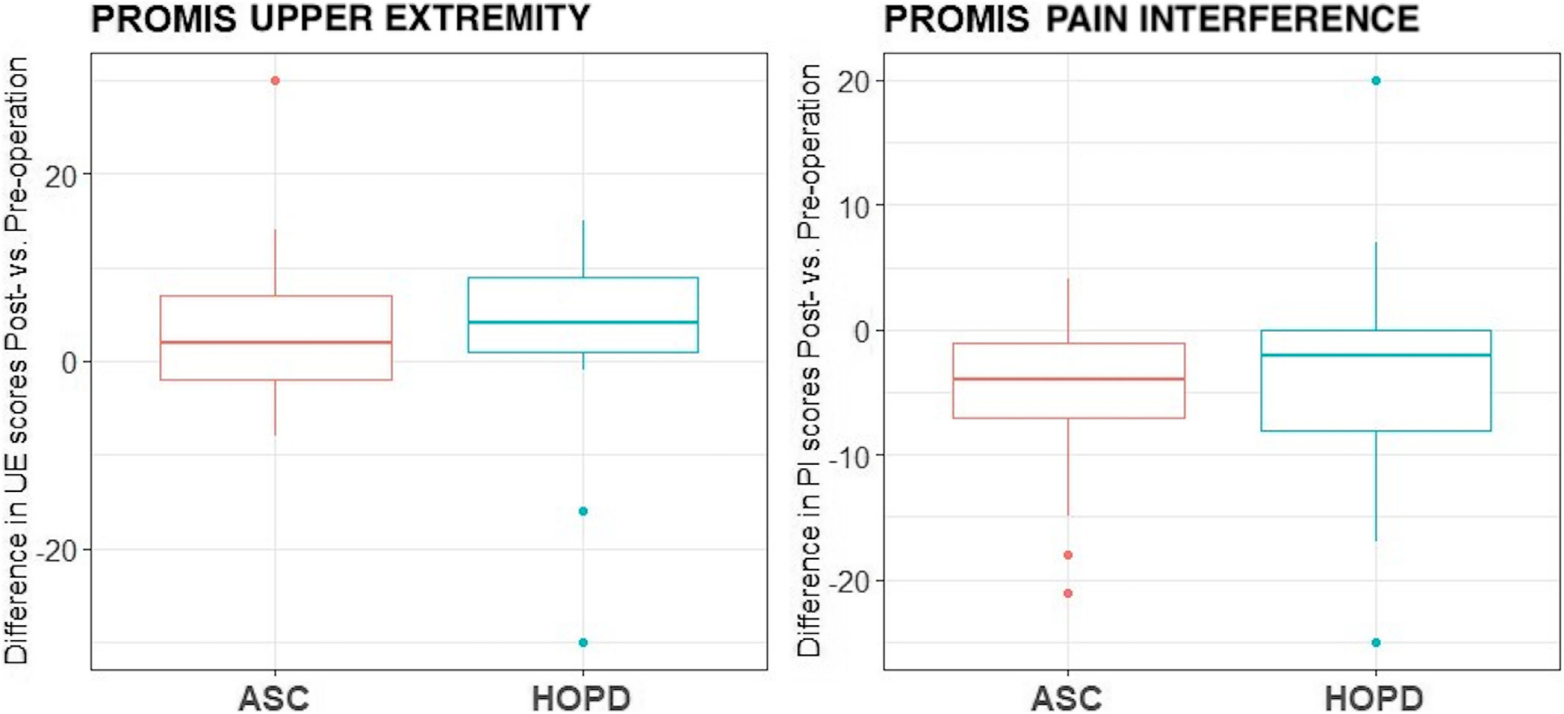
Comparison of the average differences between preoperative PROMIS UE and PROMIS PI scores for HOPD and ASC CTR surgeries and 3-month postoperative PROMIS UE and PROMIS PI scores for HOPD and ASC CTR surgeries. ∗*P* < .05. ∗∗*P* < .01. n = 25 patients per surgical modality.

There was no significant difference in the average change in PROMIS UE scores measured between cohorts from preoperative visit and 3-month postoperative visit (+3.64 ± 9.56 HOPD, +3.92 ± 8.13 ASC,; *P =* .912; Fig. 4). Similarly, there was no significant difference between average change in PROMIS PI scores between the HOPD (−3.52 ± 8.42) and ASC (−4.84 ± 6.72; *P =* .543).

The average number of life-years remaining in the HOPD and ASC cohorts was 24.1 ± 10.8 and 25.1 ± 11.9 years, respectively (*P =* .754). When multiplied by the average change in PROMIS UE scores, QALY_UE_ for the HOPD and ASC were 0.89 and 1.06, respectively. QALY_PI_ was 0.92 in the HOPD and 1.22 in the ASC.

**Figure 5:**
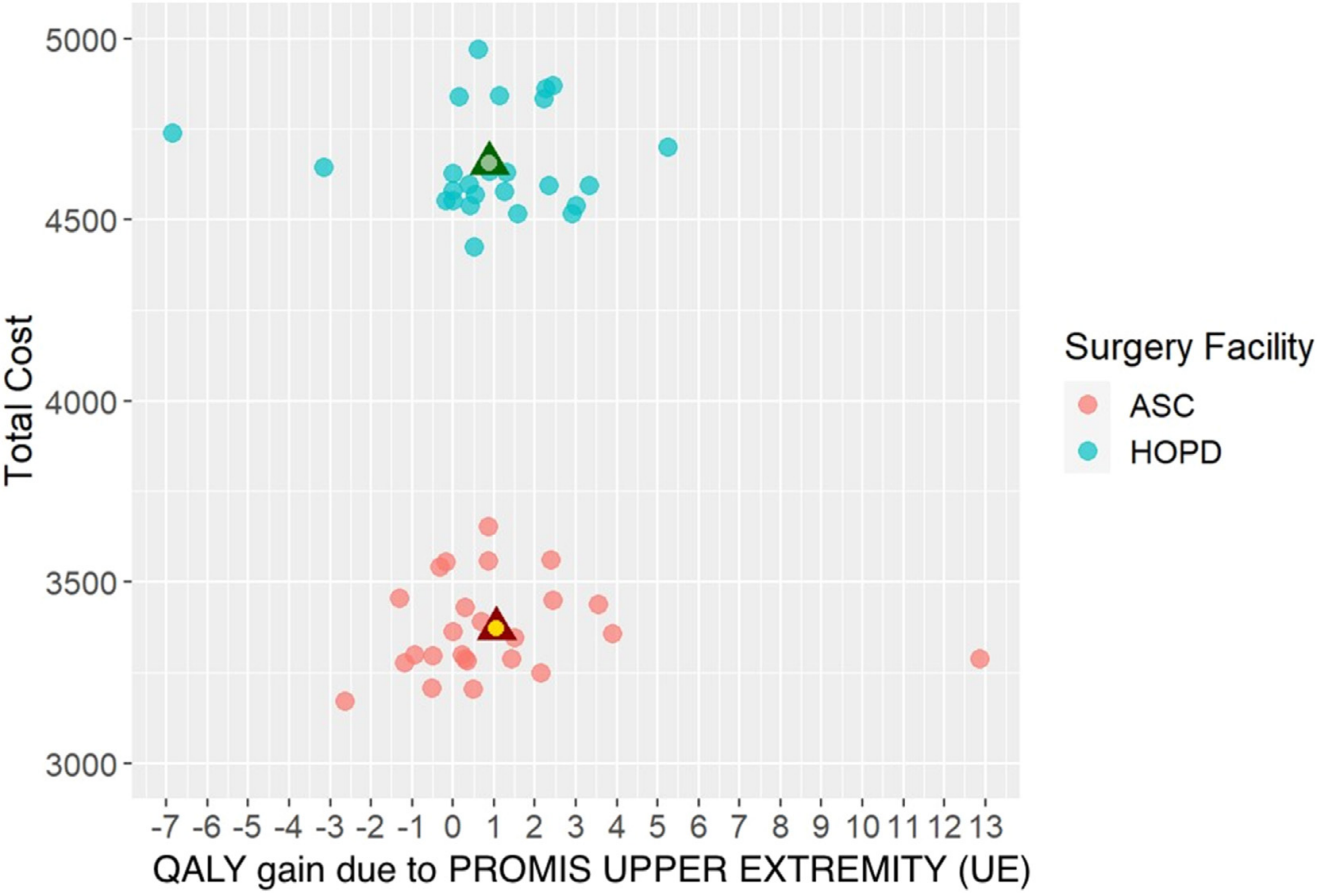
Comparison of total cost and change in preoperative PROMIS UE outcomes for HOPD and ASC CTR surgeries to 3-month postoperative PROMIS UE outcomes for HOPD and ASC CTR surgeries. Data points closer to the bottom right of the figure represent greater value provided, and data points closer to the top left of the figure represent lesser value provided. n = 25 patients per surgical modality.

**Figure 6:**
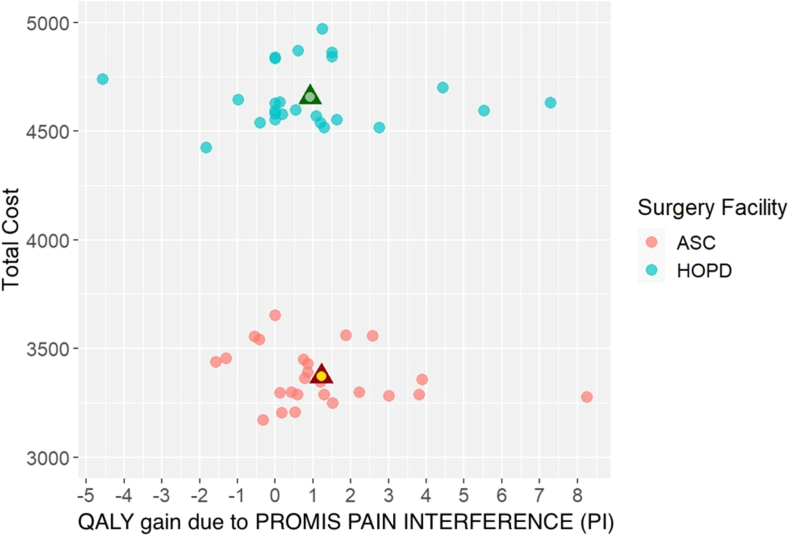
Comparison of total cost and change in preoperative PROMIS PI outcomes for HOPD and ASC CTR surgeries to 3-month postoperative PROMIS PI outcomes for HOPD and ASC CTR surgeries. Data points closer to the bottom right of the figure represent greater value provided, and data points closer to the top left of the figure represent lesser value provided. n = 25 patients per surgical modality.

The numeric Value_UE_ was lower for the ASC, indicating greater cost-effectiveness ($3,168.81/QALY; 95% confidence interval [CI]: $1,221.21; $14,100.07) ASC; $5,242.78/QALY (95% CI: $2,457.76; $29,645.61; HOPD; Fig. 5). Value_PI_ demonstrated a similar difference between surgical modalities ($2,759.90/QALY; 95% CI: $1,496.80; $5,999.89; ASC; $5,038.04/QALY; 95% CI: $2,224.85; $23,751.20; HOPD; Fig. 6). The ICER calculation for PROMIS UE comparing ASC to HOPD demonstrated a relative QALY (quality) loss with an additional $7,300.56 spent in the HOPD (95% CI: $−1,055,561.67; $−2,635.65). A similar trend was seen for PROMIS PI with an ICER equivalent to $−4,317.49/QALY (95% CI: $−1,249,338.99; $−1,586.43), also indicating a relative QALY loss while spending an additional $4,317.49.

## Discussion

In this study, we performed a patient-level value analysis using comprehensive costs in conjunction with PROMIS UE and PI outcome metrics to compare the value of performing an endoscopic CTR in an ASC and HOPD setting. Although the cost was approximately 28% lower in the ASC, we observed an insignificantly greater improvement in outcome measures relative to the HOPD, by 0.28 and 1.32 points for PROMIS UE and PI, respectively. We observed between 45% and 40% greater value when performing the CTR in the ASC compared to the HOPD, depending on whether PROMIS UE or PROMIS PI were used. This confirms our primary hypothesis that transitioning common hand procedures to lower acuity facilities can enhance value beyond just cost differential.

One of the key components in calculating value is measuring costs. We measured costs using a mixed methodology including TDABC, ABC, and claims-based data, amounting to 28% lower total costs in the ASC compared to the HOPD. Many cost-effectiveness analyses use readily accessible Medicare claims/reimbursement/payor data or ABC, although the latter to a lesser extent.^32^, ^33^, ^34^ Although these costs are a major contributor to societal health care costs, they do not inform stakeholders about how care delivery can be modified to generate greater value for patients and improve margins for health systems. Despite being recognized as a more sensitive measure of labor cost, the widespread adoption of TDABC by health care systems has yet to occur, due to the substantial resource investments necessary to implement resource-level cost accounting properly at scale.^24^ No focused study in hand surgery, to our knowledge, has conducted a cost-effectiveness analysis from a health care systems perspective by using TDABC for at least a portion of their cost accounting. The granularity into resource use afforded by TDABC provides actionable insights about process enhancement. Wang et al^11^ compared technical and professional reimbursement fees between patients undergoing CTR in an HOPD and ASC, and observed 8% greater professional fees in the ASC. Compared to our 15% lower TDABC-derived labor costs in the ASC, professional fees appear to be a poor surrogate for approximating labor and process-related costs and provide limited insight into how care delivery might be adapted to deliver better cost-effective care.

The other necessary component of value is measuring quality. Quality of care is multidimensional; some cost-effectiveness studies use objective measures, such as readmissions and complications,^8^^,^^35^ whereas others use broader outcome measures, such as the SF-6D.^32^^,^^36^ Stephens et al^37^ showed that CTR patient-reported outcomes via the Boston Carpal Tunnel Questionnaire between HOPD and procedure room (PR) were insignificantly different. However, to our knowledge, no study has compared patient-reported outcomes between the HOPD and ASC for CTR. Kronlage et al^35^ found no appreciable differences regarding quality using infection and revision rates between the HOPD and ASC for CTR surgery. Our study uses PROMIS UE and PI subjective patient-reported outcomes, with results similar to published literature using objective quality measures.

**Figure 7:**
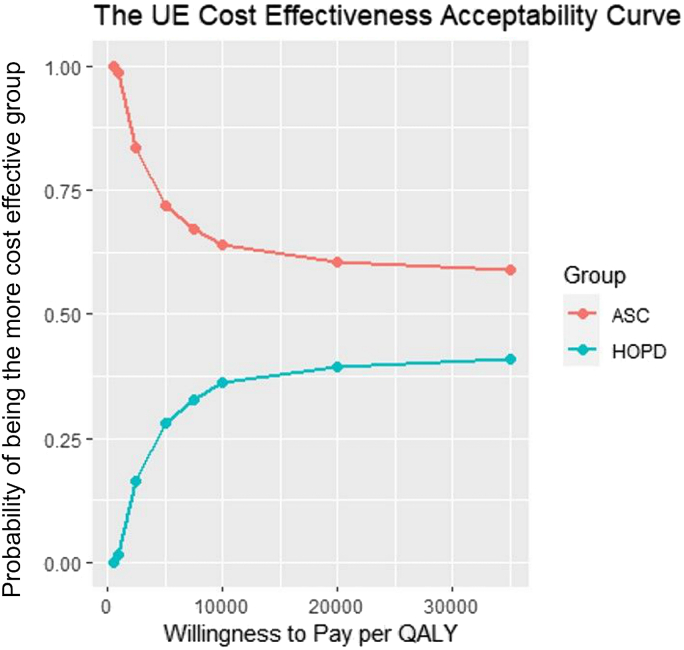
Comparison of the probability of a CTR at either surgical setting being cost-effective at increasing willingness-to-pay thresholds per QALY. Data points with a higher probability at a lower willingness to pay per QALY are more cost-effective. n = 1,000 bootstrapped samples per surgical modality.

**Figure 8:**
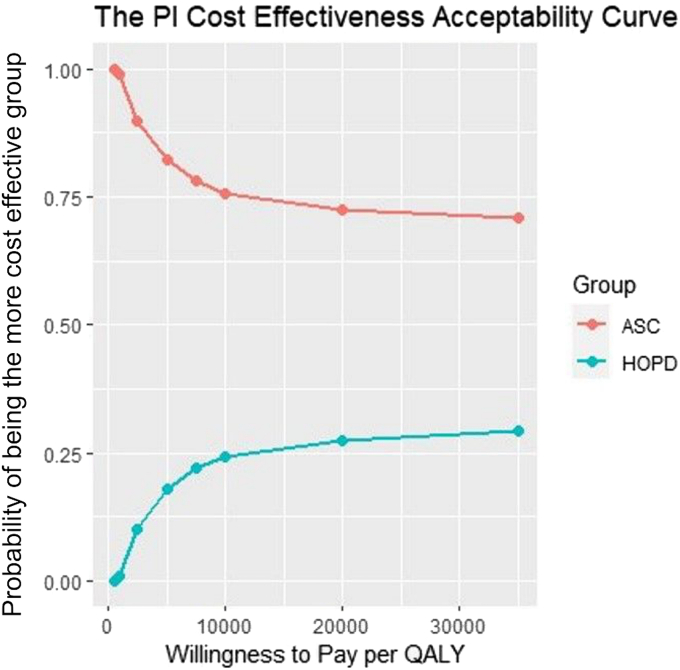
Comparison of the probability of a CTR at either surgical setting being cost-effective at increasing willingness-to-pay thresholds per QALY. Data points with a higher probability at a lower willingness to pay per QALY are more cost-effective. n = 1,000 bootstrapped samples per surgical modality.

Overall, our use of the ICER formula and CEAC demonstrated that greater value could be delivered by performing CTR in the ASC at all willingness-to-pay threshold values (Figure 7, Figure 8). Moreover, we observed a 40% to 45% difference in Value_UE/PI_ between cohorts, markedly higher than the observed 28% difference in cost. This discrepancy could be attributed to an insignificantly lower average age in the ASC by 3% and insignificantly greater outcomes in the ASC, ranging from 8% to 38%. Of note, a recent study (Rogers et al^32^) used a Markov simulation model to compare the value between an open CTR in the HOPD and PR using Medicare payment data and SF-6D patient-reported outcomes. Their simulated model demonstrated 16% lower total costs in the PR with exactly zero difference in effectiveness between the HOPD and PR, effectively assuming and attributing all differences in value to differences in cost. Markov models and other simulation-based value calculations derive their data and health-state probabilities from secondary sources. If these data do not exist or the model is constructed under the assumption that outcomes are equivalent between different surgical venues, the use and applicability of such a model may be questionable. Our study highlights the need to quantify the quality of care and incorporate it with costs to gain a true understanding of the relative value of patient-specific treatments and interventions.

Our study is not without limitations. Indirect cost calculations are one potentially limiting factor in our study. DiGioia et al illustrated the inherent difficulties with quantifying indirect costs using the TDABC methodology.^38^ As a result, studies by Akhavan et al^24^ and Palsis et al^26^ are widely used to approximate this cost category. To mitigate this limitation, we used the same proportion of DF costs to calculate the indirect costs for each of the groups being compared. Additionally, we cannot be certain of the number of patients required to have their surgeries performed in the HOPD due to comorbidities. Notwithstanding this limitation, we believe our results remain valid and enhance the practical applicability of our findings in understanding the value proposition of endoscopic carpal tunnel releases in various clinical environments.

In conclusion, we determined that performing an endoscopic CTR, using a mixed costing methodology and validated patient-reported outcomes (PROMIS UE and PI), is more cost-effective in an ASC compared to a HOPD. Moreover, the value differential we observed could not be explained entirely by the difference in cost between our cohorts. This underscores the benefit of incorporating quality metrics into discussions around health care value, along with the critical role of the ICER formula to compare the value between various treatment options. Future studies may expand upon the work in this study by using the ICER formula to determine the value of other high-volume or high-cost surgeries or comparing the value between surgical and non-surgical treatment options.

## Conflicts of Interest

The authors declare that they have no known competing financial interests or personal relationships that could have appeared to influence the work reported in this paper. The authors declare the following financial/personal relationships which may be considered as potential competing interests: Dr Charles Day has received financial compensation from Arthrex and AM Surgical for research publications not related to this study.
